# 

**Published:** 1932

**Authors:** 


### CONTENTS.

| | Page |
|---|---|
| The Long Fox Lecture: The Problem of Deafness. By A. J. M. Wright, M.B., B.S. Lond., M.B., Ch.B. Brist., F.R.C.S. | 255 |
| Prognosis in Coronary Thrombosis. By Carey F. Coombs, M.D., F.R.C.P. | 277 |
| The Effects of Lightning: with special Reference to the Nervous System. By Macdonald Critchley, M.D., F.R.C.P. | 285 |
| The Care and Management of Premature Infants. By Francis J. Hector, M.D., F.R.C.S. | 301 |
| Note on a Case of Ponto-Cerebellar Tumour in a Girl of Six Years. By A. Wilfrid Adams, M.S., F.R.C.S. | 309 |
| Provincial Medical Journals. By J. A. Nixon, C.M.G., M.D., F.R.C.P. | 311 |
| Reviews of Books | 319 |
| Editorial Notes | 325 |
| Obituary. Carey Franklin Coombs, M.D., F.R.C.P. | 326 |
| Meetings of Societies | 329 |
| The Medical Library of the University of Bristol | 332 |
| Local Medical Notes | 333 |

